# EEG Connectivity is an Objective Signature of Reduced Consciousness and Sleep Depth

**DOI:** 10.1007/s10548-025-01144-9

**Published:** 2025-09-05

**Authors:** Inken Toedt, Gesine Hermann, Enzo Tagliazucchi, Inga Karin Todtenhaupt, Helmut Laufs, Frederic von Wegner

**Affiliations:** 1 Department of Neurology, Christian-Albrechts University, Arnold-Heller-Strasse 3, 24105 Kiel, Germany; 2 Present Address: Institute of Sexual Medicine & Forensic Psychiatry and Psychotherapy, Christian- Albrechts University, Schwanenweg 24, 24105 Kiel, Germany; 3 Latin American Brain Health Institute (BrainLat), Universidad Adolfo Ibañez, Santiago, Chile; 4 Facultad de Ciencias Exactas y Naturales, Departamento de Física, Instituto de Física Interdisciplinaria y Aplicada (INFINA), Universidad de Buenos Aires, CONICET - Universidad de Buenos Aires, Inga Karin, Buenos Aires, Todtenhaupt, Argentina; 5 School of Biomedical Sciences, University of New South Wales (UNSW), Wallace Wurth Building, Kensington, NSW 2052 Australia

**Keywords:** Electroencephalography, Coherence, Functional connectivity, Phase coupling, Sleep stage classification, Machine learning, Consciousness

## Abstract

Different levels of reduced consciousness characterise human sleep stages at the behavioural level. On electroencephalography (EEG), the identification of sleep stages predominantly relies on localised oscillatory power within distinct frequency bands. Several theoretical frameworks converge on the central significance of long-range information sharing in maintaining consciousness, which experimentally manifests as high functional connectivity (FC) between distant brain regions. Here, we test the hypothesis that EEG-FC reflects sleep stages and hence changes in consciousness. We retrospectively investigated sleep EEG recordings in 14 participants undergoing all stages of non-rapid eye movement (NREM) sleep. We quantified FC with six phase coupling metrics and used the FC coefficients between electrode pairs as features for a gradient boosting classifier trained to distinguish between sleep stages. To characterise FC during each stage of NREM sleep, we compared these metrics regarding their classification accuracy and analysed the ranked feature importance across all electrode pairs. We observed frequency-specific differences in FC between sleep stages for all metrics except the imaginary part of coherence. Alpha coupling decreased from wake to sleep stages N1 and N2, whereas delta coupling increased in deep sleep (N3). FC-based sleep classifiers yielded 51% (phase locking index) to 73% (phase locking value) classification accuracy. Distributed FC patterns in the alpha band ranked highest in terms of feature importance. In a limited sample of 14 subjects, we demonstrated that FC computed from phase information changes significantly across sleep stages. The finding that EEG phase patterns are indicative of sleep stages supports the hypothesis that long-range and spatially distributed phase coupling within frequency bands, especially within the alpha band, is an electrophysiological correlate of consciousness across sleep stages.

**Supplementary Information:**

The online version contains supplementary material available at 10.1007/s10548-025-01144-9.

## Key Points

1. EEG functional connectivity allows classification of the non-REM sleep stages W, N1-N3.

2. Machine learning classifiers can identify sleep stages based on phase coupling patterns alone.

3. Alpha and delta band phase coupling are key features of sleep-related changes of consciousness.

4. Feature importances are widely distributed and do not exhibit sleep stage-specific spatial patterns.

**Supplementary Information:**

The online version contains supplementary material available at 10.1007/s10548-025-01144-9.

## Introduction

During their studies of electroencephalographic activity during sleep, in 1935 Loomis and colleagues reported “random”, “saw tooth”, “trains of waves” and spindles, and ‘that the individual rhythms arise in different regions of the cortex, but that the gradual change of type from “trains” to “random” is dependent on a change connected with the cortex as a whole’ (Loomis et al. 1935). Despite this early appreciation of the global nature of brain activity changes during sleep, current standard rules for sleep stage classification mainly rely on the detection of local oscillations in specific frequency bands recorded over occipital, central and frontal brain regions (Iber et al. 2007; Rechtschaffen and Kales 1968). In particular, interactions between distant brain regions such as those described by functional or effective connectivity measures (Friston 1994, 2011) are not considered during the process of visual sleep stage scoring. Yet, studies based on EEG and other neuroimaging techniques (such as functional magnetic resonance imaging [fMRI]) reveal changes in long-range information sharing associated with sleep (Sämann et al. 2011; Spoormaker et al. 2010; Tagliazucchi et al. 2013b; Tagliazucchi et al. 2013; Tagliazucchi and van Someren 2017), which are sufficient for the algorithmic classification of sleep stages with 75% accuracy (Tagliazucchi and Laufs 2014).

In polysomnography, of which EEG is the main component, a sequence of discrete and distinct stages defines the human sleep cycle (Iber et al. 2007; Rechtschaffen and Kales 1968). The transition from wakefulness (W) to the first sleep stage (N1, nomenclature of the American Academy of Sleep Medicine [AASM]) is characterised by a shift from EEG dominated by an occipital alpha rhythm (8–12 Hz) to low-amplitude, mixed frequency activity (4–7 Hz) with phase reversing discharges over the central regions, known as vertex sharp waves. Other sleep grapho-elements (SGE), namely sleep spindles (bursts of 12–16 Hz oscillations) and K-complexes (large amplitude deflections) signal the onset of N2 sleep, which is followed by a stage characterised by global low frequency (< 4 Hz) oscillations (N3 sleep). One cycle from W to N3 lasts around 90 min and is followed by the first episode of rapid eye movement (REM) sleep. This is characterized by a restoration of high frequency EEG activity and is often accompanied by reports of vivid dreams after awakenings (Bosinelli 1995).

One of the most interesting aspects of sleep is the reduction of conscious awareness (Iber et al. 2007; Tagliazucchi et al., 2013a; Behrens, et al., 2013; Tononi et al. 2016), understood as the capacity to experiment and report first-person subjective experiences (Dehaene and Naccache 2001). In contrast to the electrophysiological parameters recorded with polysomnography, an objective assessment of different degrees of conscious awareness during sleep has not been established yet. However, serial awakening paradigms suggest that falling asleep is characterised by reduced consciousness, and the combination with EEG indicates that subjective experience correlates with the onset of local EEG rhythms in posterior brain regions (Siclari et al. 2017). Accordingly, sleep serves as a useful reversible and non-invasive model to investigate the neurophysiological basis of conscious awareness in healthy individuals (Bosinelli 1995; Hobson 2009; Hobson and Friston 2012; Picchioni et al. 2013). While theoretical models of consciousness converge on the relevance of long-range information sharing (King et al. 2013; Luppi et al. 2019) which might be reflected in EEG phase-coupling metrics, connectivity metrics are not part of current sleep scoring rules. Using the common definition of functional connectivity (FC) as a measure of statistical temporal interdependency between a neurophysiological index measured in spatially separated brain regions (Friston 1994), we hypothesized that electroencephalographic FC metrics might be indicative of long-range information sharing during NREM sleep.

Current neuroimaging studies indicate that brain FC fluctuates across different sleep stages, reflecting varying levels of consciousness and brain network organisation (Tagliazucchi, von Wegner, Morzelewski, Brodbeck et al. 2012; Tagliazucchi and van Someren 2017). Alterations in FC, particularly in slower frequency bands, align with the global reduction in consciousness during sleep (Tagliazucchi et al., 2013b). Although some frequency bands and coupling metrics have been studied in the past (Tanaka et al. azu 1999; Lee et al. 2019; Huang et al. 2021), a comprehensive assessment across different FC metrics and multiple frequency bands over the NREM sleep stages is lacking.

In this study, we test the relationship between EEG-FC and states of reduced consciousness. We hypothesize that EEG-FC depends on sleep stage and that machine learning classifiers can be trained to classify NREM sleep stages based on phase relationships. We calculated EEG-FC based on phase coupling metrics for each sleep stage and subsequently applied supervised machine learning (ML) for the classification of sleep stages from the FC features. To probe to what extent EEG amplitude information - in addition to phase alone - contributes to the classification accuracy, we used purely phase-based metrics on the one hand and metrics in which both phase and spectral power are considered, on the other.

## Materials and methods

### Participants and Data Acquisition

EEG recordings of 14 healthy right-handed subjects (21–26 yrs, mean age 22.4 ± 1.6 yrs; 7 male) were included in this retrospective study. We included subjects who had reached each NREM sleep stage according to the classification of the AASM (Iber et al. 2007) for at least two minutes (Table 1). The dataset is part of a simultaneous EEG-fMRI study on sleep physiology and has been explored and validated in earlier reports (Brodbeck et al. 2012; Jahnke et al. 2012). The results presented here are based on the analysis of the EEG data only. To balance the data set used for machine learning, we only included subjects for whom all four sleep stages (W, N1-N3) were available. As phase calculations are sensitive to noise, we removed sleep epochs with residual muscle or movement artefacts. This reduced the size of the data set compared to the one used in (Brodbeck et al. 2012).

### EEG Recording, Preprocessing and Sleep Stage Classification

EEG was acquired during simultaneous EEG-fMRI recordings for 52 min as detailed previously (Brodbeck et al. 2012; Jahnke et al. 2012). Briefly, 30 EEG channels (BrainAmp MR+,BrainAmp ExG; Brain Products, Gilching, German), a subset of the standard 10–10 layout, were initially sampled at 5 kHz, referenced to FCz, and corrected for MR gradient and ballistocardiogram artefacts offline. EEG signals were then band-pass filtered to 1–40 Hz (IIR filter as implemented in the Brain Vision Analyzer 2.0, Brain Products GmbH, Gilching, Germany), downsampled to 250 Hz and an average reference was applied.

Sleep scoring followed the rules defined by the AASM (Iber et al. 2007). For this report, particular attention was paid to the selection of continuous, artefact-free EEG segments that could be clearly assigned to one of the NREM sleep stages according to AASM. The visual exclusion of artefacts contains a source of human bias which was controlled by using several assessors. One artefact-free data segment of each NREM sleep stage per subject was manually selected for further analysis. The resulting EEG segments had a minimum length of 100 s with a mean duration of 204 s (Table 1). All participants reached all NREM sleep stages (W, N1, N2 and N3) with epochs of at least 100 s per sleep stage.

**Table 1:** Subject and EEG characteristics. For each subject, the length of the selected NREM sleep stage (W, N1, N2, N3) segment is given in seconds (s). Bottom row (Average): mean ± sd age; fn/mn: number of female/male subjects, mean EEG segment length in seconds

			EEG segment length (s)			
ID	Age (yrs)	Gender	W	N1	N2	N3
*1*	*24*	*f*	*180*	*188*	*183*	*181*
*2*	*21*	*m*	*183*	*123*	*182*	*181*
*3*	*21*	*f*	*210*	*210*	*210*	*210*
*4*	*21*	*m*	*120*	*120*	*120*	*120*
*5*	*23*	*m*	*270*	*270*	*270*	*270*
*6*	*22*	*m*	*300*	*300*	*300*	*300*
*7*	*26*	*m*	*181*	*185*	*183*	*180*
*8*	*23*	*m*	*120*	*120*	*120*	*120*
*9*	*24*	*m*	*270*	*270*	*270*	*270*
*10*	*21*	*f*	*240*	*240*	*240*	*240*
*11*	*20*	*f*	*150*	*150*	*150*	*150*
*12*	*23*	*f*	*150*	*150*	*150*	*150*
*13*	*21*	*f*	*300*	*300*	*300*	*300*
*14*	*24*	*f*	*184*	*180*	*181*	*181*
*Average*						
	*22.4 ± 1.64*	*fn = 7*	*204*	*203*	*204*	*204*
		*mn = 7*				

The EEG segment length in s divided by 10 corresponds to the number of windows included in the classification approach

### Functional Connectivity Measures

To quantify EEG-FC, we calculated six phase coupling parameters for band-pass filtered EEG signals from the four canonical EEG frequency bands, and for each sleep stage (delta: 1–3.5 Hz, theta: 4.5–7 Hz, alpha: 8–12 Hz, and beta: 13–30 Hz). Compared to the standard frequency band definition (Feyissa and Tatum 2019), we introduced gaps of 1 Hz between adjacent frequency bands to reduce the overlap of phase information at the frequency boundaries between band-pass filters.

EEG segments representing one sleep stage were band-pass-filtered into the four frequency bands using a 6-th order, zero-phase digital Butterworth filter (25 db/octave). The analytic phase signals were obtained from the Hilbert transform of the band-pass-filtered signals.

For each band-pass filtered EEG signal $\:x\left(t\right)$, representing one EEG sensor, with Hilbert transform $\:{x}_{H}\left(t\right)$, the complex analytic signal $\:x\left(t\right)+i{x}_{H}\left(t\right)=A\left(t\right)\times\:exp\left(-i\varphi\:\left(t\right)\right)$ defines the analytic amplitude $\:A\left(t\right)$ and phase $\:\varphi\:\left(t\right)$. From these, the cross spectra $\:{S}_{ij}={A}_{i}\left(t\right){A}_{j}\left(t\right)\times\:exp\left(-\varDelta\:{\varphi\:}_{ij}\left(t\right)\right)$ and power spectra $\:{S}_{ii}={A}_{i}^{2}\left(t\right)$ of channels $\:i$ and $\:j$ are derived, their phase difference being $\:\varDelta\:{\varphi\:}_{ij}\left(t\right)={\varphi\:}_{i}\left(t\right){-\varphi\:}_{j}\left(t\right)$.

EEG segments representing a single sleep stage were segmented into epochs of 10 s and six phase coupling metrics were computed over each epoch. The six coupling parameters were calculated in the time domain ($\:A\left(t\right)$, $\:\varphi\:\left(t\right)$), instead of using the frequency domain definition of coherence, an approach detailed in Stam et al. 2007 and Bruña et al. 2018.

1. Coherence (COH) (Mormann et al. 2000):

$$\:COH\:=\:\frac{\left|\langle{S}_{ij}\rangle\right|}{\sqrt{\langle{S}_{ii}\rangle\langle{S}_{jj}\rangle}}$$

2. The imaginary part of coherency (iCOH) (Nolte et al. 2004):

$$\:iCOH\:=Im\left(\frac{\langle{S}_{ij}\rangle}{\sqrt{\langle{S}_{ii}\rangle\langle{S}_{jj}\rangle}}\right)\:$$

3. The phase-locking value (PLV) (Lachaux et al. 1999):

$$\:PLV\:=\left|\langle\frac{{S}_{ij}}{\left|{S}_{ij}\right|}\rangle\right|\:$$

4. The corrected imaginary part of the phase locking value (ciPLV) (Bruña et al. 2017):

$$\:ciPLV\:=\frac{\left|\langle Im\left(\frac{{S}_{ij}}{\left|{S}_{ij}\right|}\right)\rangle\right|}{\sqrt{1-{\left|\rangle Re\left(\frac{{S}_{ij}}{\left|{S}_{ij}\right|}\right)\rangle\right|}^{2}}}\:$$

5. The phase lag index (PLI) (Stam et al. 2007):

$$\:PLI\:=\left|\langle sign\left(Im\left({S}_{ij}\right)\right)\rangle\right|\:$$

6. The weighted phase lag index (wPLI) (Vinck et al. 2011):

$$\:wPLI\:=\frac{\left|\langle Im\left({S}_{ij}\right)\rangle\right|}{\langle\left|Im\left({S}_{ij}\right)\right|\rangle}\:$$

All averages $\langle\cdot\rangle$ denote time averages across a 10 s epoch.

COH and iCOH depend on phase and amplitude components of the EEG signal and have been criticized for this property in studies aiming at source localization and “true” phase connectivity at the level of neural generators, or sources (Bastos and Schoffelen 2016). While it is true that amplitude and phase information are confounded to a certain extent, it is not clear whether this will have a positive or negative effect on machine learning (ML) classification. iCOH only considers the imaginary part of the complex-valued coherency metric, thereby eliminating instantaneous correlations between two channels, which are attributed to volume conduction effects. As iCOH still depends on the signal amplitude, strong volume conduction effects can increase the iCOH denominator and lead to a reduced range of iCOH values (Stam et al. 2007).

The remaining metrics use amplitude-normalized signals. PLV averages unit length vectors whose argument (angle) is the phase difference $\:\varDelta\:{\varphi\:}_{ij}\left(t\right)$ (Lachaux et al. 1999). Values are close to one if the phase difference is constant and approaches zero as phase differences become inconsistent. ciPLV eliminates the instantaneous correlations (volume conduction effects) contained in PLV and introduces a correction factor accounting for the magnitude of the real part of $\:{S}_{ij}$ (Bruña et al. 2017).

In contrast to PLV, PLI only considers the asymmetry of the phase lag distribution in terms of the sign of $\:\varDelta\:{\varphi\:}_{ij}\left(t\right)$ (lag vs. lead) (Stam et al. 2007). The presence of noise can randomly push the PLI components across the $\:\varDelta\:{\varphi\:}_{ij}=0$ border, an effect accounted for in the wPLI definition.

In summary, two of the metrics we study are sensitive to volume conduction effects (COH, PLV), and two are sensitive to both phase and amplitude components (COH, iCOH). If volume conduction effects aid the classifier, COH/PLV will outperform iCOH/ciPLV, respectively. Likewise, COH/iCOH will outperform the other metrics if signal amplitudes help with classification. PLI is not expected to perform better than wPLI as the former is more sensitive to noise.

All analyses were implemented in the Python 3.8 programming language (Van Rossum and Drake 2009).

### Statistical Analysis and Machine Learning Approach

For each FC parameter, the Shapiro Wilk test was used to investigate whether the values deviated from a normal distribution. We therefore calculated one test for each FC parameter and frequency band across all subjects and all sleep stages (*N* = 1137; number of EEG segments across all subjects). Since they were not normally distributed (for all FC parameters *p* < 0.001), non-parametric approaches were used for all statistical analyses. To test for overall differences between sleep stages within each connectivity parameter and frequency band, we used Friedman tests. To control for the family-wise error rate (FWER) across the multiple Friedman tests (6 connectivity parameters × 4 frequency bands = 24 tests), Bonferroni correction was applied, resulting in an adjusted significance threshold of α = 0.05/24 = 0.00208. Only Friedman tests with p-values below this corrected threshold were considered significant (Supplementary Table 1). In case of overall significant differences, we used post-hoc Nemenyi tests that correct for a familywise error, implemented in the SciPy (Virtanen et al. 2020) and Scikit-posthocs (Terpilowski 2019) packages, to further detect significant differences between each pair of sleep stages. For the Nemenyi tests, we used a p-value of 0.05 for significance within each FC parameter. The selection of the independent variables was based on the further processing of the data in the machine learning approach, in which we trained a classifier separately for each FC parameter that contributed one value per EEG segment in each of the four frequency bands. Importantly, the primary focus of our study lies in the Gradient Boosting approach, which operates independently of these initial statistical tests.

The ML task used the sleep stage labels W, N1, N2, N3 as the target variable, and classified these from the FC coefficients. We trained six independent classifiers, one for each coupling parameter (COH, iCOH, PLV, ciPLV, PLI, wPLI). The feature vectors contained 4 × 435 = 1740 coefficients. This resulted from the 435 unique off-diagonal entries of the 30 × 30 symmetric connectivity matrices (30 EEG channels), and the fact that we had one connectivity matrix for each of the four frequency bands (delta, theta, alpha, beta). Each sample represented the value of the given coupling parameter averaged over a 10 s window of EEG data, without overlap.

We chose the gradient boosting classifier (GBC) as implemented in the Scikit-learn package for Python (Pedregosa et al. 2012). Using a 5-fold cross validation scheme we tested all hyperparameter combinations defined by: learning rate = [0.005, 0.01, 0.05, 0.1, 0.15], number of estimators = [250, 500, 750, 1000, 1250, 1500], and maximum depth = [3, 4, 5, 6, 7].

To this end, we divided our cohort into 30 different 80/20 splits, each split consisting of 11 subjects for training and 3 subjects for testing. We trained and optimized a GBC for each of the 30 training data sets (11 subjects each), and tested it on the remaining 3 subjects, respectively. This procedure led to 30 optimized classifiers. We report the mean accuracies, confusion matrices, and feature importance scores for the sleep stage classification averaged over all 30 GBCs.

As an additional analyses, we applied the same ML approach to spectral power features (30 EEG channels x 4 frequency bands) to gauge the relative importance of phase and power for classification. The results are shown in the supplement (Supplementary Fig. 3).

## Results

### Sleep stage-dependent Differences in FC Parameters

**Fig. 1:**
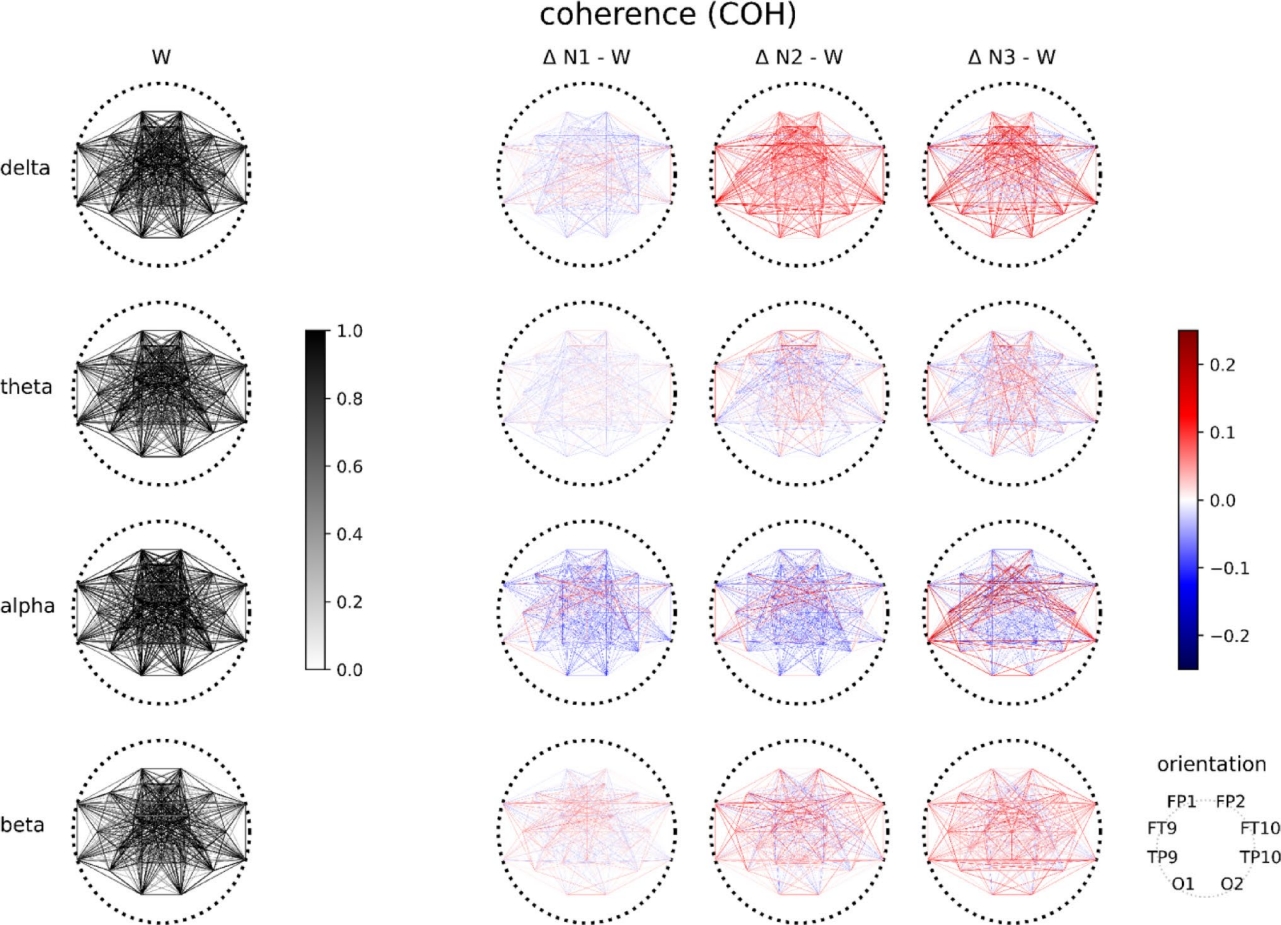
Coherence (COH) in the alpha frequency band decreases during the transition from wakefulness (W) to sleep (N1, N2), while COH in the delta frequency band increases during sleep (N2, N3). The left column displays segment-averaged absolute COH values between pairs of electrodes for each frequency band (delta, theta, alpha, beta) during wakefulness (W). The subsequent columns illustrate the changes in COH between the respective sleep stages (N1, N2, N3) and W. A decrease in COH during sleep is represented in blue, while increased COH is shown in red. The plot shows the average COH across all subjects

Figure 1 displays coherence values (COH) for each frequency band during wakefulness (W) and the differences compared to each of the three sleep stages (N1, N2, N3). It demonstrates global changes in FC during sleep characterized by an increase in COH in the delta frequency band and a decrease in the alpha frequency band as sleep deepens. These effects are consistent across all FC measures. The corresponding plots for iCOH, PLV, ciPLV, PLI, and wPLI are shown in Supplementary Fig. 1

**Fig. 2:**
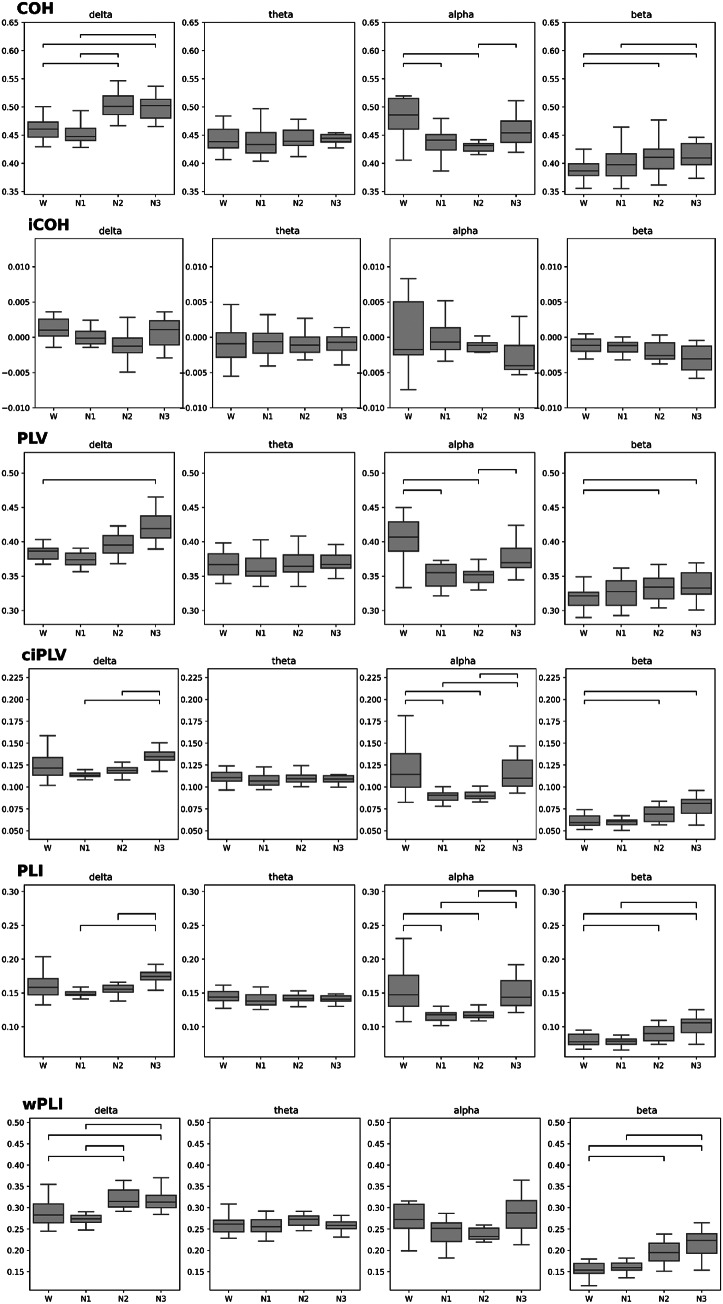
Averaged FC parameters (subject-level, *N* = 14) for each EEG frequency band (delta: 1–3.5 Hz, theta: 4.5–7 Hz, alpha: 8–12 Hz, beta: 13–30 Hz). Horizontal bars indicate a significant difference between the respective sleep stages (*p* < 0.05, Nemenyi post-hoc test)

To test for differences in FC between sleep stages we calculated Friedman tests and post-hoc Nemenyi tests for each connectivity parameter and frequency band separately. Figure 2 shows FC parameters averaged over all electrode pairs and time segments within a given sleep stage. We identified significant differences between sleep stages in the delta, alpha and beta band for all FC parameters except for iCOH, where no significant differences were found, and for wPLI, where significant differences were only found in the delta and beta band. Overall, sleep stage discrimination depended on the frequency band, the connectivity parameter, and sleep depth. Prominently, phase coupling measures in the alpha band (COH, PLV, ciPLV, PLI) distinguished between W and N1 or N2. Delta band coupling (COH, PLV, wPLI) distinguished well between W and N3, and similar results were obtained for beta band coupling (COH, PLV, ciPLV, PLI, wPLI). Detailed numerical results of the Friedman and post-hoc tests are given in Supplementary Table 1.

**Fig. 3:**
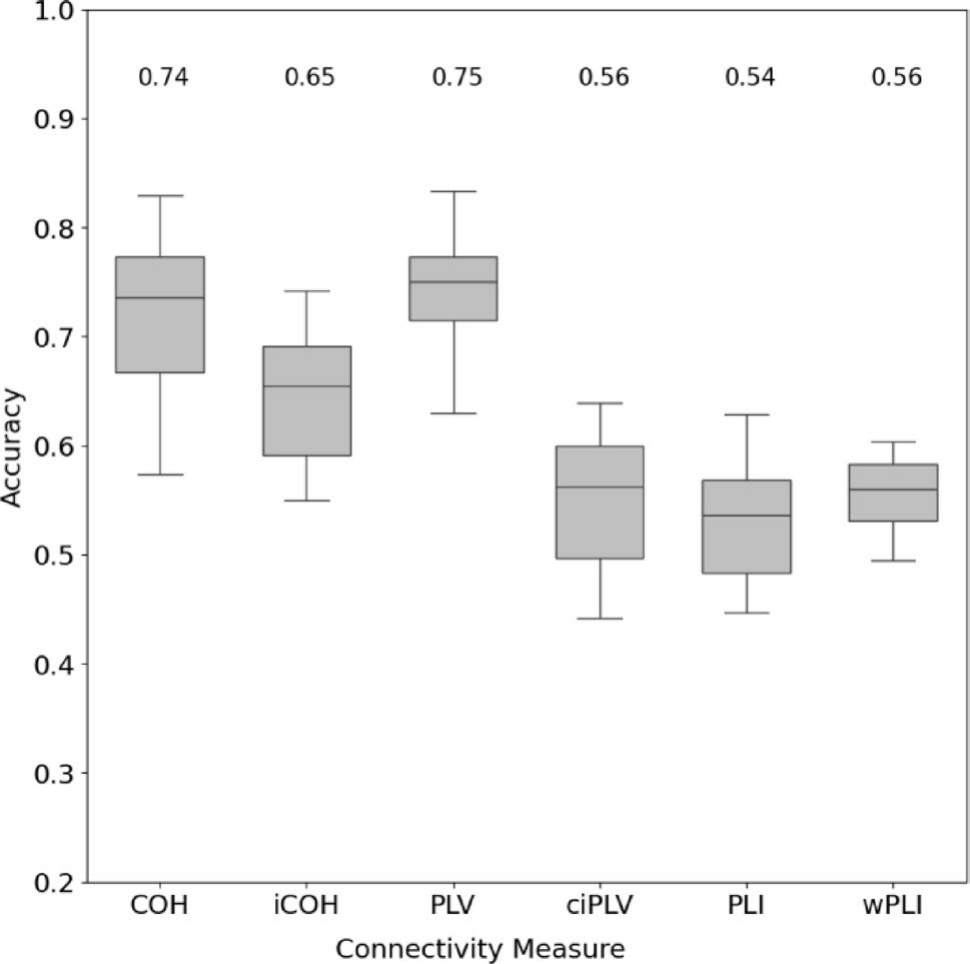
Classification accuracy distributions over 30 Gradient Boosting Classifiers (GBC), each based on a different 80/20 subject split. Boxplots: median (horizontal bar), first and third quartiles (lower and upper box edge), minimum and maximum values (whiskers). Medians are shown numerically above each box. Note that the y-axis scaling starts at 0.2. An ANOVA on the FC parameters yielded a significant result (F_5,174_ = 77.36, *p* < 0.001)

### Sleep Stage Classification Based on FC

**Fig. 4:**
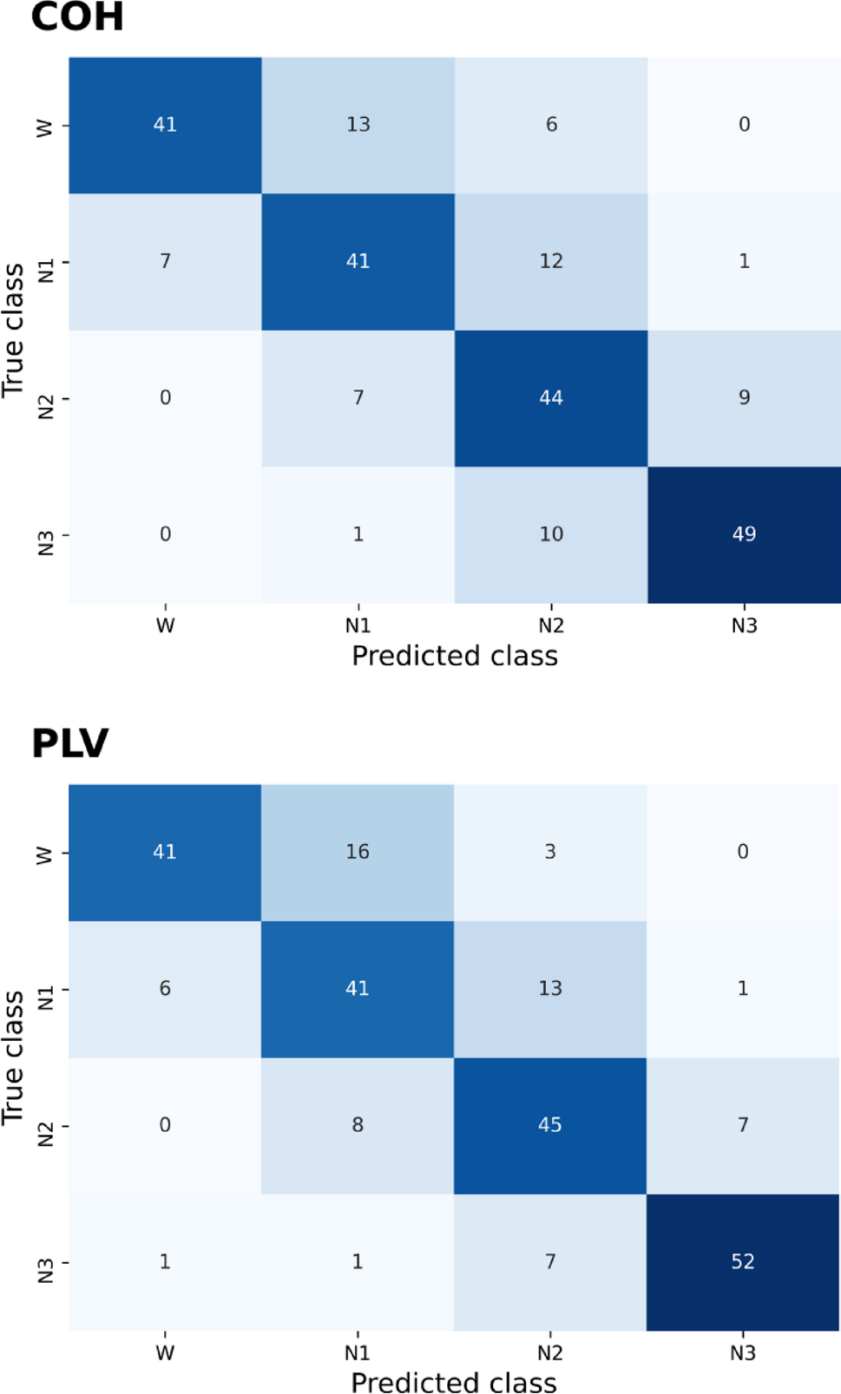
Confusion matrices for classification results based on the phase coupling metrics COH (upper panel) and PLV (lower panel), averaged over 30 train-test splits. The matrices display the number of correctly classified sleep stage epochs on the diagonal. Off-diagonal elements represent incorrect classifications. AASM-based sleep scoring was set as ground truth

Averaging the classification accuracy values over the 30 GBCs for each connectivity parameter resulted in a classification accuracy above the chance level of 0.25 (equal mean length of all sleep stages, see Table 1), for all phase coupling metrics (Fig. 3). COH and PLV resulted in the highest accuracies of 0.74 and 0.75, respectively. Tukey HSD post hoc tests showed no significant difference in classification accuracy between COH and PLV (*p* = 0.76), but both were significantly better than the remaining four FC measures (*p* < 0.001). We therefore focus on the discussion of these two metrics from here on, however, the results for the other four parameters can be found in the Supplementary Figures S1, S2, S3. The confusion matrices for each metric (Fig. 4) show that the COH- and PLV-based classifiers differentiated best between sleep stages W-N2 and W-N3. Conversely, classification errors occurred especially between W and N1, and between N2 and N3, i.e. adjacent stages within the sleep cycle. Stages visually classified as N1 (AASM) were often misclassified as W, and N2 (AASM) as N1. We considered manual classification based on AASM rules as ground truth.

### Feature Importance Scores

**Fig. 5:**
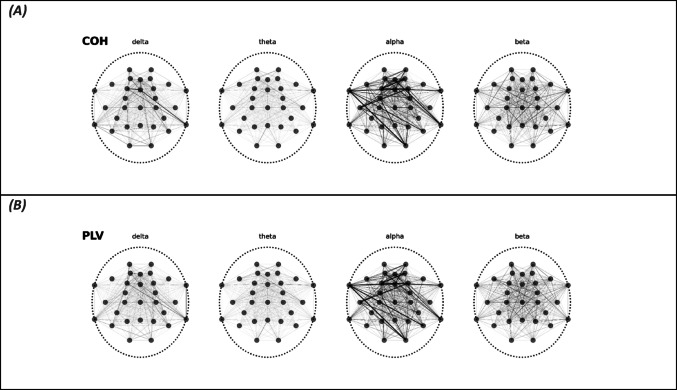
Mean feature importance scores for (A) COH- and (B) PLV-based gradient boost classifiers (GBC). Line width reflects the feature importance of each EEG sensor pair. Note that feature importance scores are normalized and sum up to 1 in A and B, respectively, to allow visual comparison of line widths between frequency bands

Based on the optimised classification results, we visualised the learned feature importance scores for each frequency band topographically in Fig. 5. Note that feature importance values are normalised and sum up to 1. The results are shown in Fig. 5 and are very similar for COH and PLV. Classifications based on COH and PLV, respectively, were both largely driven by alpha band phase coupling. Of note, alpha coupling across widespread cortical regions contributed to classification accuracy as opposed to that of predominantly those posterior regions, where alpha activity is most prominent and on which AASM rules are based. We observed similar spatially distributed patterns for the other frequency bands. In terms of feature importance, beta and delta frequency band coupling ranked second and third behind alpha coupling, while the theta band contributed the least discriminatory features.

## Discussion

We investigated the relationship between EEG-derived brain functional connectivity and varying degrees of reduced conscious awareness during sleep. This was motivated by theoretical models of consciousness and our own previous fMRI results proposing alterations in long-range functional connectivity as a fundamental mechanism underlying different levels of consciousness (King et al. 2013; Luppi et al. 2019; Tagliazucchi et al., 2013a; Tagliazucchi et al. 2012; Tagliazucchi, von Wegner, Morzelewski, Brodbeck et al. 2012; Tagliazucchi and van Someren 2017).

We found phase coupling values (COH, PLV, ciPLV, PLI, wPLI) that varied with sleep stage (W, N1, N2 and N3) and frequency band (delta, theta, alpha, beta), but we did not observe sleep stage-specific spatial patterns. Optimised classifiers based on phase information demonstrated classification accuracies ranging from 54 to 75%, all of which were significantly higher than chance level.

### Distributed Alpha and Delta Phase Coupling Is Most Relevant for Sleep Stage Discrimination

Wakefulness significantly differed from N1 and N2 in terms of phase coupling (coherence) within the alpha frequency band (Fig. 1). Discrimination between deep sleep (N3) and the stages of lighter sleep relied on coupling in the delta, alpha, and beta frequency bands (Fig. 2, Supplementary Table 1). The substantial feature importance of coupling in the alpha-frequency band, highest during W, is in line with earlier results on sleep stage-dependent coherence (Achermann and Borbély 1998) and so is our observation of increasing delta coupling with deepening sleep (Huang et al. 2021; Tanaka et al. 1999; Torres-Herraez et al. 2022) and unconsciousness in particular (Lee et al. 2019). It is noteworthy that the criteria for visually scoring sleep stages W and N3 are also primarily based on neural oscillations in the alpha and delta frequency spectra (Iber et al. 2007; Rechtschaffen and Kales 1968). Phase coupling evades easy visual assessment and hence related scoring rules (AASM) cannot take it into account. The latter rely on amplitude criteria, to which some of our metrics are blind: The two FC metrics that exhibited the highest accuracy included both amplitude-sensitive (COH) and amplitude-agnostic (PLV) metrics. This demonstrates that phase coupling reflects relevant functional information independent of oscillatory amplitude, which suggests its capacity in conditions in which neurobiological information is not primarily and specifically encoded in oscillatory amplitude.

Theta band coherence features contributed less to sleep stage discrimination. This might be the consequence of only a few subjects - already by visual inspection - exhibiting clear oscillatory theta activity in N1. Increased theta power in N1 often appears as a broad elevation in power spectral density plots, rather than displaying a resonance-like peak as observed for alpha oscillations. This lack of narrow-band theta oscillations might explain the lower feature importance of phase coupling values in the theta band. In a similar fashion, visual scoring criteria for W vs. N1 are less precise than N1 vs. N2 criteria for example. N2 sleep stage markers such as sleep spindles are distinct features with a clear spectral mark in the beta band (amplitude and coherence, compare Figs. 1, 2 and 5 and Supplementary Figs. 1 and 3). This relative lack of distinct spectral features in N1 might have impacted the reliability of W vs. N1 classification both at the visual (gold standard, training data) and the feature (theta coherence) levels.

### Phase Coupling Is Spatially Distributed

We did not observe clear spatial patterns of the studied phase coupling metrics in any frequency band or sleep stage. These spatially widely distributed phase coupling features contrast with visual sleep scoring rules that emphasize the importance of occipital alpha and frontal theta oscillations (Iber et al. 2007). While some studies have proposed a “posterior hot zone” as a correlate of consciousness using EEG and other techniques (Boly et al. 2017; Siclari et al. 2017; Koch et al. 2016), our findings did not reveal an occipital dominance in alpha coupling during wakefulness. Bouchard et al. (2020) investigated sleep stage-dependent differences in EEG coherence, and - as this study - found spatially widespread changes (Bouchard et al. 2020). In contrast to these widely distributed EEG-FC patterns (Fig. 3), fMRI with its refined spatial sampling reveals significant reorganisation within cortical (Tagliazucchi et al. 2012a; Tagliazucchi and Laufs 2014; Tagliazucchi and van Someren 2017) and cortico-subcortical FC modules, including the thalamus and cerebellum (Tagliazucchi et al. 2013). However, these changes cannot be expected to appear in surface EEG connectivity maps, first because EEG records primarily cortical activity, and second because EEG sensor signals exhibit high correlation due to volume conduction (Bhavsar et al. 2018; Cohen and Tsuchiya 2018). Nevertheless, subcortical reorganisation can indirectly impact surface EEG signals by influencing global cortical activity patterns. For example, thalamic synchronisation of cortical oscillations is expected to have significant effects on EEG-observable neuronal oscillations and to affect most EEG sensors, rather than generating regional effects only (Neske 2016). Considering all pairwise phase coupling values might thus make the classification procedure sensitive to subcortical brain activity and improve its performance. Following theories of consciousness, this sensitivity might be particularly useful in the study of consciousness.

### Volume conduction-sensitive Metrics Outperform amplitude-sensitive Metrics

As discussed in the previous paragraph, volume conduction is a possible explanation for distributed FC patterns and is often considered a nuisance in surface EEG connectivity analyses (Nunez et al. 1997; van den Broek et al. 1998; Vinck et al. 2011). Therefore, research groups have developed metrics that try to eliminate spurious coupling due to volume conduction (Bruña et al. 2017; Nolte et al. 2004; Hardmeier et al. 2014; Stam et al. 2007; Vinck et al. 2011). In our study, FC metrics sensitive to volume conduction (COH, PLV) resulted in higher classification accuracy than those that aim to reduce instantaneous correlations (iCOH, ciPLV). Furthermore, metrics that retain some information about the signal amplitude in the FC coefficient, i.e. COH (Mormann et al. 2000) and iCOH (Nolte et al. 2004), did not achieve higher accuracies than those that discard signal amplitude.

Among the FC metrics tested on our dataset, phase coupling metrics affected by volume conduction (COH, PLV) yielded the most accurate sleep stage classification. A possible explanation is that sleep spindles and K-complexes, key features for AASM-based sleep scoring (Iber et al. 2007), originate from sources and oscillators located deep within the brain. As discussed further above, projections from these deep sources can lead to correlated signals at multiple surface sensors (Fernandez and Lüthi 2020; Fogel and Smith 2011; Jahnke et al. 2012; Roth et al. 1956; Wennberg 2010). Eliminating these biologically grounded correlations algorithmically might negatively impact classifier accuracy.

### Comparison with Other Sleep Classifiers

The primary aim of our study was not a technical one but biologically motivated. We did not aim to introduce yet another sleep scoring algorithm, but to explore to which extent EEG phase coupling features reflect sleep stage and the degree of consciousness related to these stages. The classification accuracy achieved with this approach and on our relatively small data set is outperformed by recent deep learning techniques using transformer designs (Brandmayr et al. 2022), but also with convolutional and other network architectures (Perslev et al. 2021; Mousavi et al. 2019; Phan et al. 2019; Supratak et al. 2017; Supratak and Guo 2020; Tsinalis et al. 2016a; 2016b). For example, Supratak‘s DeepSleepNet analysed a single bipolar EEG channel and achieved a maximum accuracy of 82% (Supratak et al. 2017), slightly higher than the earlier autoencoder approach by Tsinalis et al. (Tsinalis et al. 2016) who achieved accuracies of 79% and 74.8% using convolutional networks (Tsinalis et al. 2016b). This is slightly more accurate than our best performing solution (PLV, 73%). The more recent TinySleepNet (Supratak and Guo 2020) achieved 85.4% accuracy on the same data set, thereby performing slightly better than SeqSleepNet+‘s 85.2% (Phan et al. 2019) and SleepEEGNet’s 84% accuracies (Mousavi et al. 2019).

A direct comparison of our results with the ones listed above is not possible as, for example, the Sleep-EDF data (Kemp et al. 2000) analysed by several of the groups referenced above consists of two EEG channels only and includes significant noise. This raises the question why our PLV-based sleep staging performs worse despite more channels and perhaps less noise? A possible answer is that EEG signals unrelated to brain activity could still help to classify sleep stages efficiently. Electrophysiological recordings of sleep contain a mixture of physiological and neurobiological signals, including patterns of muscle activity, eye movements, breathing patterns and movement in addition to alterations in consciousness. Such “artefacts” could boost the classifier performance compared to when only cortically generated EEG activity is considered (Tagliazucchi et al. 2012a, b).

### Phase Coupling as a Marker of Reduced Consciousness

Our results suggest a possibly wider use of phase coupling information for the assessment of states of reduced consciousness other than sleep. Sleep stage classification, as standardised by the AASM, uses feature combinations that are highly specific for human sleep, for example vertex sharp waves, sleep spindles, and K-complexes (Fernandez and Lüthi 2020; Iber et al. 2007; Roth et al. 1956). These sleep-specific features with defined spectral and topographic signatures are absent in encephalopathies where the EEG is often characterized by general EEG slowing without electrographic transients (Sutter et al. 2015). Phase coupling patterns are a candidate for the characterization of such encephalopathic states with reduced consciousness and altered long-range connectivity, in analogy to the connectivity changes previously explored with fMRI across different sleep stages (Tagliazucchi et al. 2013). For example, the prominence of low-frequency oscillations observed in N3 is not exclusive to sleep but may also serve as an indicator of reduced consciousness in broader contexts (Banks et al. 2020; Fernandez et al. 2020; Höller et al. 2014; Moody et al. 2021). Höller et al. (2014) reported EEG FC patterns that could discriminate different states of reduced consciousness (vegetative and minimally conscious state) from one another and from the healthy state. Slow oscillations are believed to modulate cortico-cortical interactions (Staresina et al. 2023) that are essential for information integration, and possibly for consciousness. However, EEG delta activity per se is not a safe indicator of reduced consciousness, and the simultaneous assessment of other EEG properties (e.g. entropy) has been suggested (Frohlich et al. 2021).

Hence, phase coupling features might contribute to a common reference parameter set when comparing sleep with other conditions of altered consciousness. Sleep data is relatively easy to obtain with minimal artefacts and at a large scale and could be used to train machine learning classifiers to be later applied to other altered states of consciousness as a “consciousness detector”. However, the success of such a detector hinges on training the model with features that predominantly capture changes in consciousness per se, distinct from EEG properties only indirectly linked to consciousness such as primary vigilance or effects of different cognitive aspects, which are potentially confounding factors. Identifying the common features among classifiers for conditions involving altered consciousness per se may represent the next significant step in this direction.

### Future Directions

Given the widespread availability of EEG, our approach could be further evaluated alongside classical sleep scoring methods in sleep laboratories (Bouchard et al. 2020; Migliorelli et al. 2019), during the administration of anaesthetics (Artoni et al. 2022; Chennu et al. 2016; Moody et al. 2021; Purdon et al. 2013), and across a broad spectrum of neurological conditions, such as epilepsy syndromes (Lüders et al. 2014), traumatic brain injury, and neurodegenerative diseases. In these contexts, patient care could benefit from the quantification of alertness and consciousness. Applications include assessing the effects and side effects of CNS-active drugs and determining fitness to drive in epilepsy and other conditions.

## Conclusion

EEG functional connectivity, assessed through phase coupling measures, exhibits sleep stage-specific changes that enable sleep stage identification via machine learning classifiers. The highest classification accuracies were achieved using the phase locking value (PLV) and the coherence (COH) metrics. Our findings support the hypothesis that long-range and distributed phase coupling within specific frequency bands, particularly the alpha band, serves as a robust indicator of sleep-related consciousness levels. This phase-driven method complements amplitude-driven measures, suggesting its potential as a tool for quantifying different degrees of consciousness in conditions beyond sleep.

## Supplementary Information

Below is the link to the electronic supplementary material.

Supplementary Material 1

## Data Availability

No datasets were generated or analysed during the current study.
