# Correction to: EEG Connectivity Is an Objective Signature of Reduced Consciousness and Sleep Depth

**DOI:** 10.1007/s10548-026-01175-w

**Published:** 2026-02-09

**Authors:** Inken Toedt, Gesine Hermann, Enzo Tagliazucchi, Inga Karin Todtenhaupt, Helmut Laufs, Frederic von Wegner

**Affiliations:** 1 Department of Neurology, Christian-Albrechts University, Arnold-Heller-Strasse 3, 24105 Kiel, Germany; 2 Present Address: Institute of Sexual Medicine & Forensic Psychiatry and Psychotherapy, Christian- Albrechts University, Schwanenweg 24, 24105 Kiel, Germany; 3 Latin American Brain Health Institute (BrainLat), Universidad Adolfo Ibañez, Santiago, Chile; 4 Facultad de Ciencias Exactas y Naturales, Departamento de Física, Instituto de Física Interdisciplinaria y Aplicada (INFINA), Universidad de Buenos Aires, CONICET - Universidad de Buenos Aires, Inga Karin, Buenos Aires, Todtenhaupt, Argentina; 5 School of Biomedical Sciences, University of New South Wales (UNSW), Wallace Wurth Building, Kensington, NSW 2052 Australia

**Correction to: Brain Topography (2025) 38:63**

10.1007/s10548-025-01144-9

In the original version of this article, the given and family name of first author Dr. Toedt were incorrectly structured.

The original article has been corrected.

