# Supplementary material for: EEG Connectivity is an Objective Signature of Reduced Consciousness and Sleep Depth
Source: Brain Topogr. 2025 Sep 5;38(6):63. doi: 10.1007/s10548-025-01144-9 (PMC12413340; doi:10.1007/s10548-025-01144-9)
Supplement: Supplementary file 1 — Supplementary Material 1 [file 10548_2025_1144_MOESM1_ESM.docx]

**Supplement**

*Supplementary table 1. Results of Friedman and Nemenyi post-hoc tests. Friedman test p-values were considered statistically significant if below the Bonferroni-corrected threshold of 0.00208, controlling for the family-wise error rate (FWER) across all tests (printed in bold). Nemenyi post hoc tests used a significance level of p < 0.05.*

|  | **freq. band** | **Chi square** | **P value** | **W – N1** | **W – N2** | **W – N3** | **N1 – N2** | **N1 – N3** | **N2 – N3** |  |
| --- | --- | --- | --- | --- | --- | --- | --- | --- | --- | --- |
| **COH** | Delta | 22.89 | **<0.0001** | 0.9000 | **0.0070** | **0.0114** | **0.0014** | **0.0025** | 0.9000 |  |
|  | Theta | 1.80 | 0.6149 |  |  |  |  |  |  |  |
|  | Alpha | 18.26 | **0.0004** | **0.0180** | **0.0010** | 0.5457 | 0.7100 | 0.3736 | **0.0419** |  |
|  | Beta | 18.86 | **0.0003** | 0.9000 | **0.0419** | **0.0010** | 0.1702 | **0.0070** | 0.6279 |  |
| **PLV** | Delta | 29.91 | **<0.0001** | 0.2268 | 0.4608 | **0.0042** | 0.3736 | 0.1246 | 0.9000 |  |
|  | Theta | 6.09 | 0.1075 |  |  |  |  |  |  |  |
|  | Alpha | 23.23 | **<0.0001** | **0.0014** | **0.0010** | 0.4608 | 0.9000 | 0.1246 | **0.0278** |  |
|  | Beta | 16.03 | **0.0011** | 0.6278 | **0.0278** | **0.0014** | 0.3736 | 0.0617 | 0.7922 |  |
| **iCOH** | Delta | 11.40 | 0.0097 |  |  |  |  |  |  |  |
|  | Theta | 1.71 | 0.6337 |  |  |  |  |  |  |  |
|  | Alpha | 3.69 | 0.2975 |  |  |  |  |  |  |  |
|  | Beta | 8.91 | 0.0305 |  |  |  |  |  |  |  |
| **ciPLV** | Delta | 17.06 | **<0.0001** | 0.1702 | 0.7100 | 0.2268 | 0.7100 | **0.001** | **0.0180** |  |
|  | Theta | 0.77 | 0.8563 |  |  |  |  |  |  |  |
|  | Alpha | 22.89 | **<0.0001** | **0.0014** | **0.0025** | 0.900 | 0.900 | **0.0070** | **0.0114** |  |
|  | Beta | 20.06 | **0.0002** | 0.7922 | **0.0419** | **0.001** | 0.2947 | 0.0770 | 0.4610 |  |
| **PLI** | Delta | 17.74 | **<0.0001** | 0.2947 | 0.7100 | 0.1246 | 0.8746 | **0.0010** | **0.0070** |  |
|  | Theta | 0.77 | 0.8563 |  |  |  |  |  |  |  |
|  | Alpha | 24.86 | **<0.0001** | **0.0010** | **0.0025** | 0.9000 | 0.9000 | **0.0025** | **0.0070** |  |
|  | Beta | 20.06 | **0.0002** | 0.7922 | **0.0419** | **0.0010** | 0.2947 | **0.0070** | 0.4608 |  |
| **wPLI** | Delta | 19.46 | **0.0002** | 0.8743 | **0.0278** | **0.0419** | **0.0025** | **0.0042** | 0.9000 |  |
|  | Theta | 2.31 | 0.5098 |  |  |  |  |  |  |  |
|  | Alpha | 14.23 | 0.0026 |  |  |  |  |  |  |  |
|  | Beta | 26.83 | **<0.0001** | 0.7922 | **0.0042** | **0.0010** | 0.0617 | **0.0014** | 0.6278 |  |

*
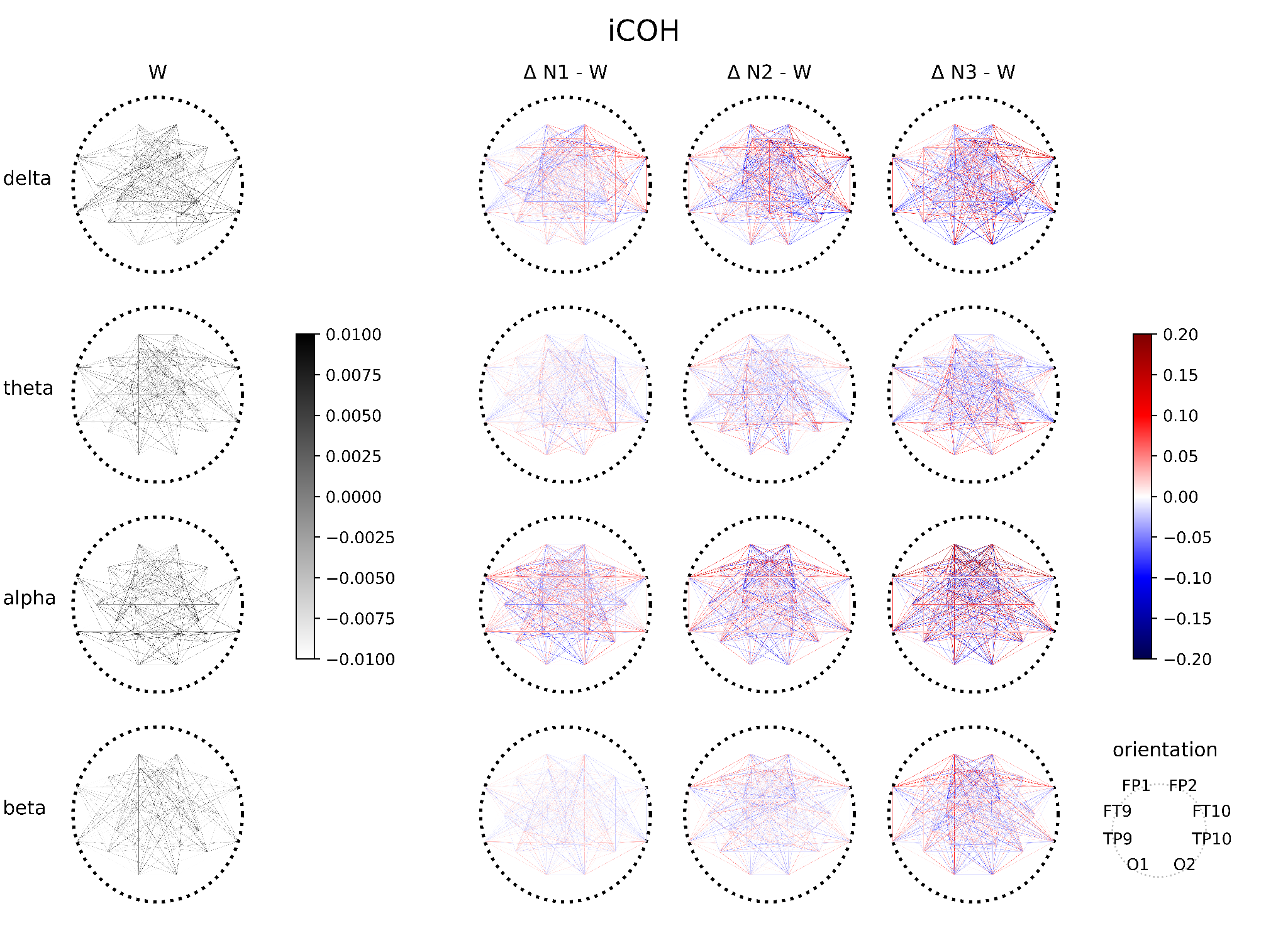
*

*
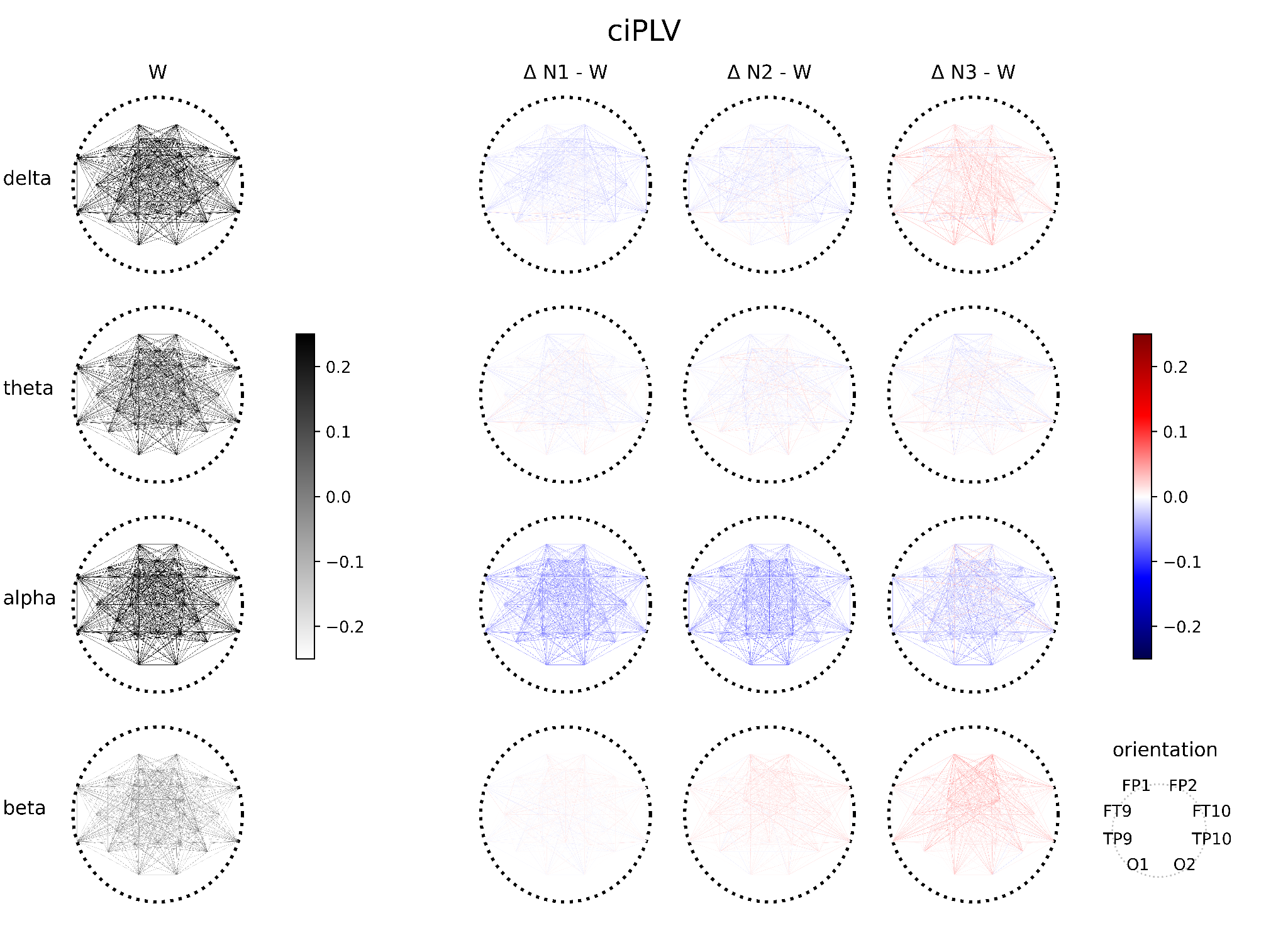
*

*
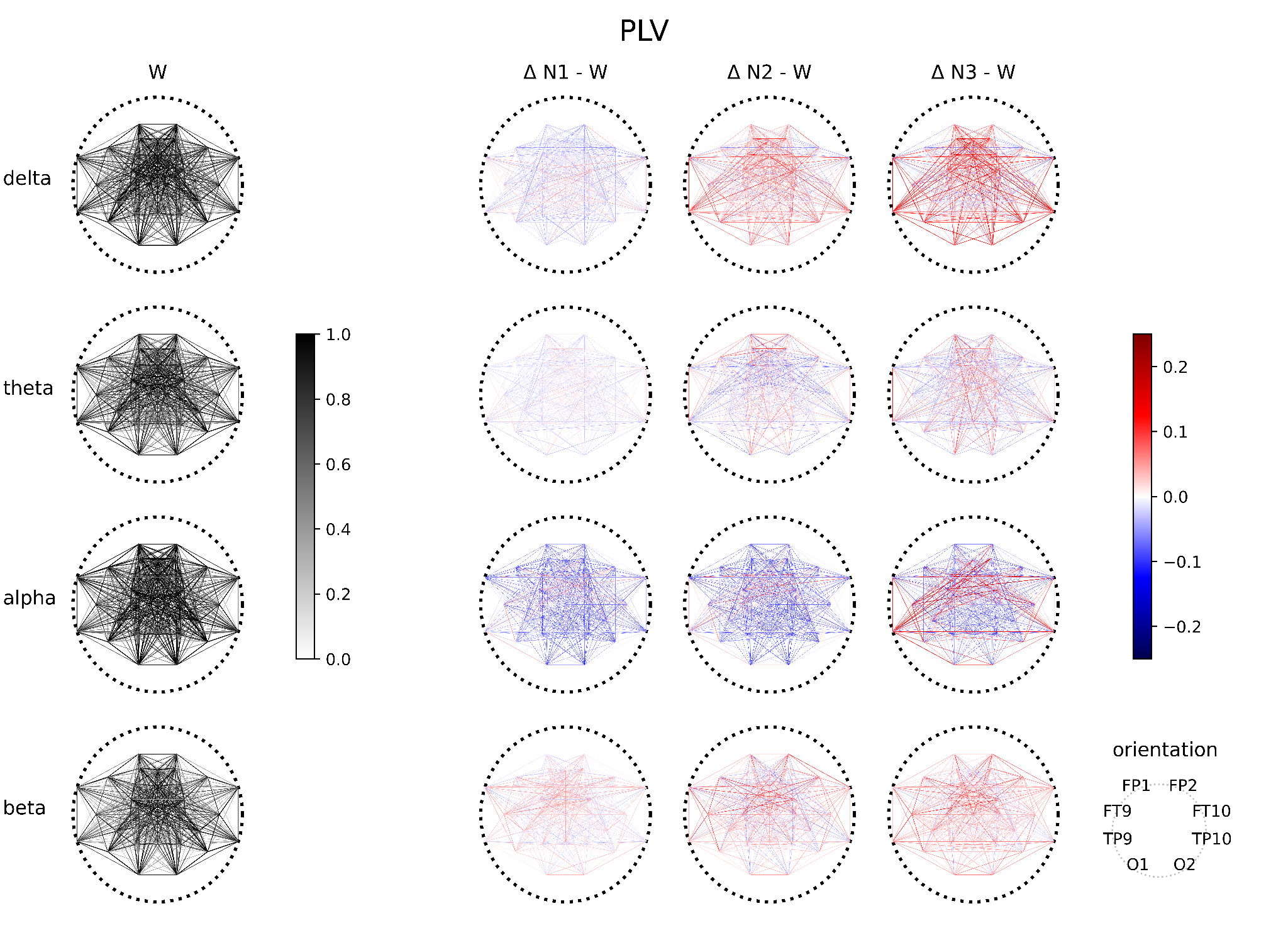
*

*
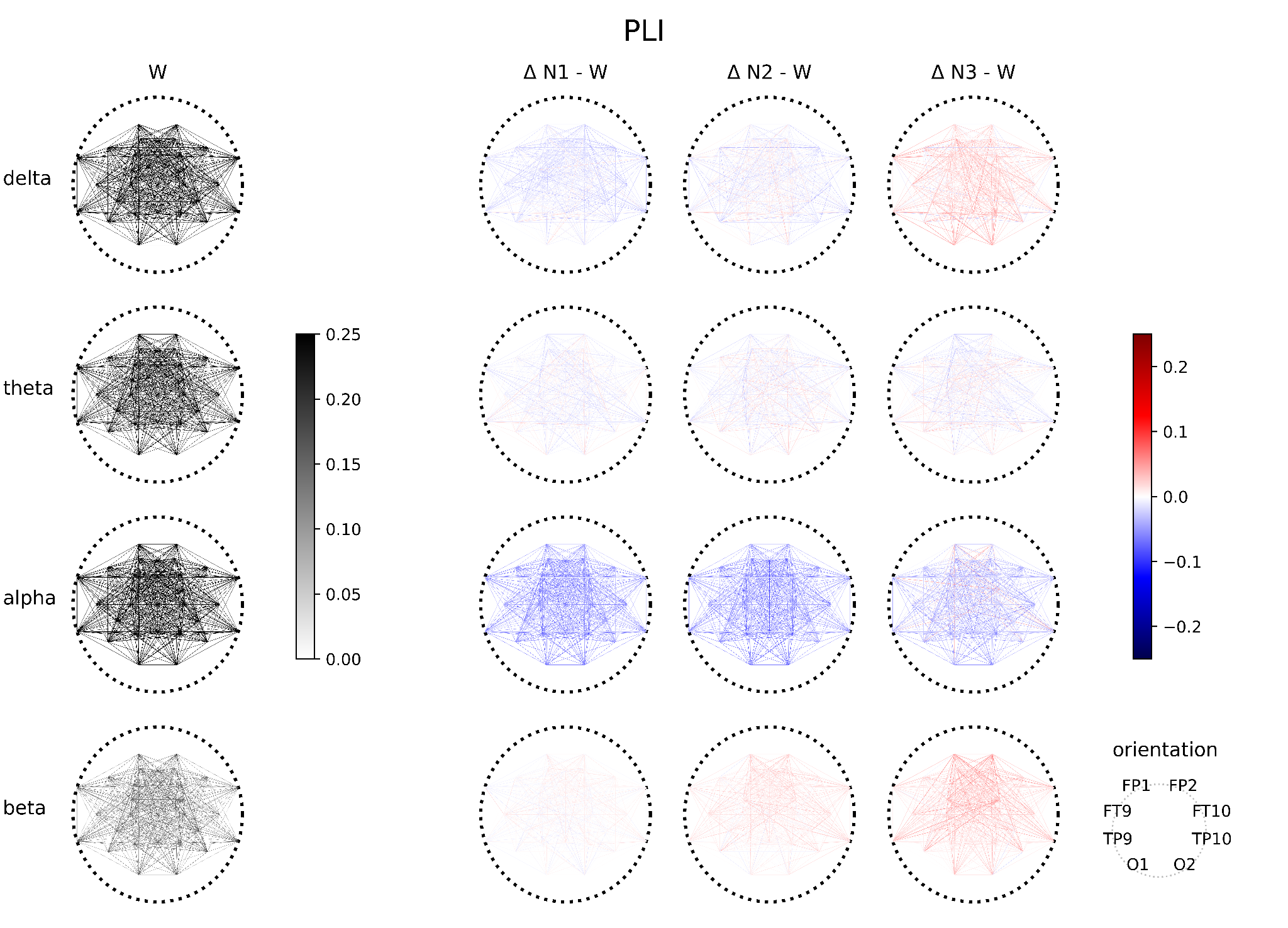
*

*
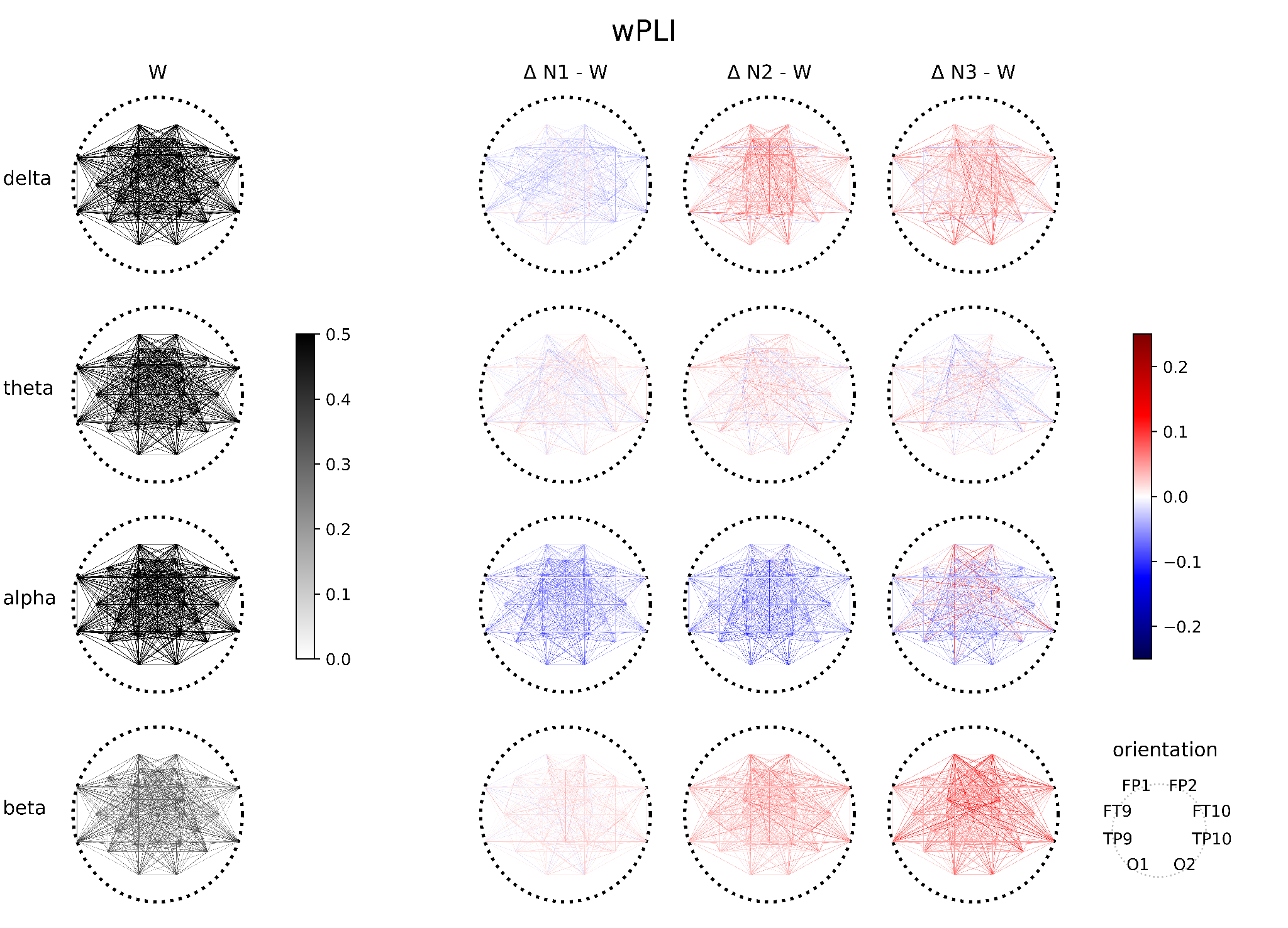
*

***Figure S1.*** *The left column displays segment-averaged iCOH, ciPLV, PLV, PLI, WPLI values between pairs of electrodes for each frequency band (delta, theta, alpha, beta) during wakefulness (W). The subsequent columns illustrate the changes between the respective sleep stages (N1, N2, N3) and W. A decrease during sleep is represented in blue, while an increase is shown in red.*

| 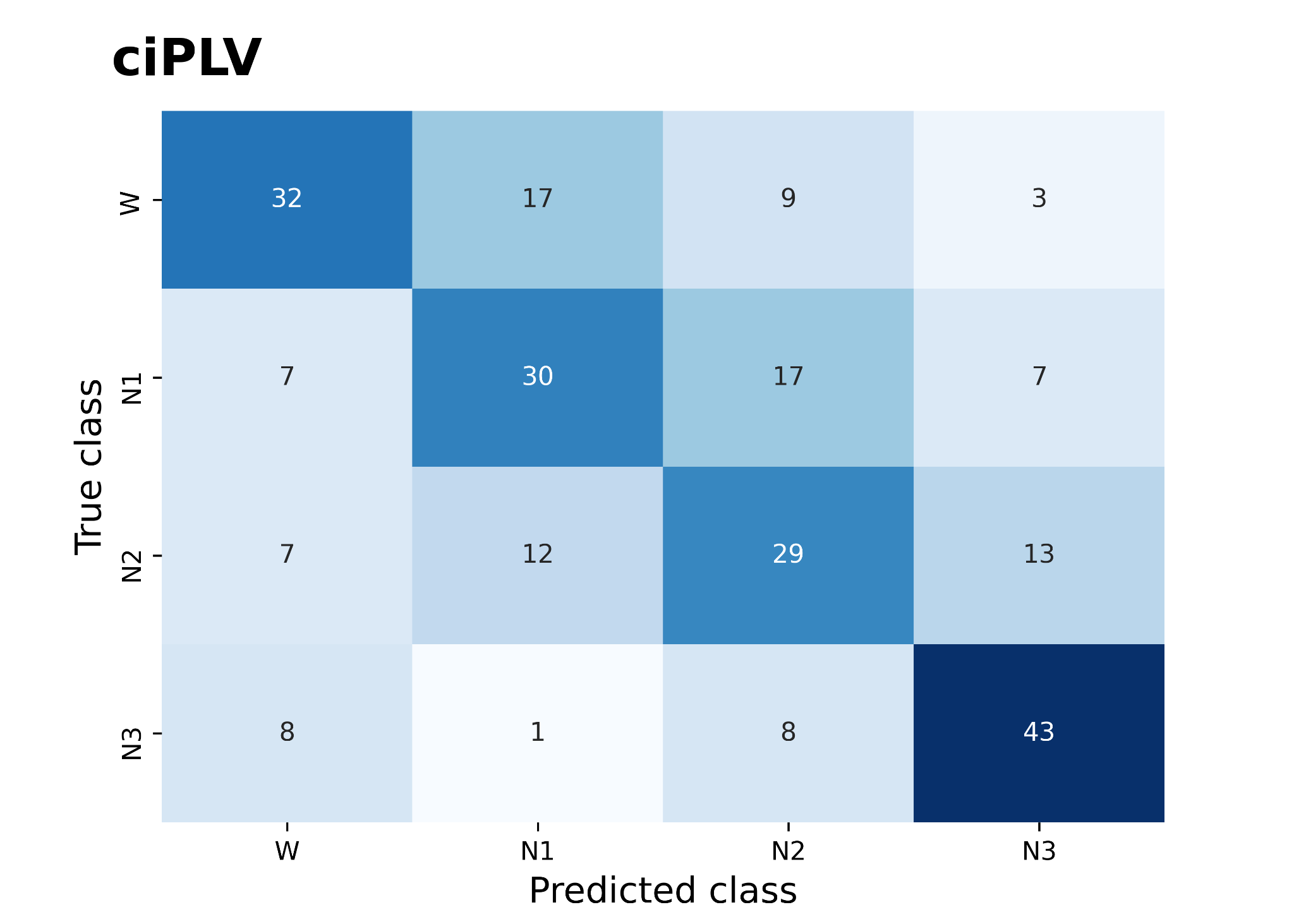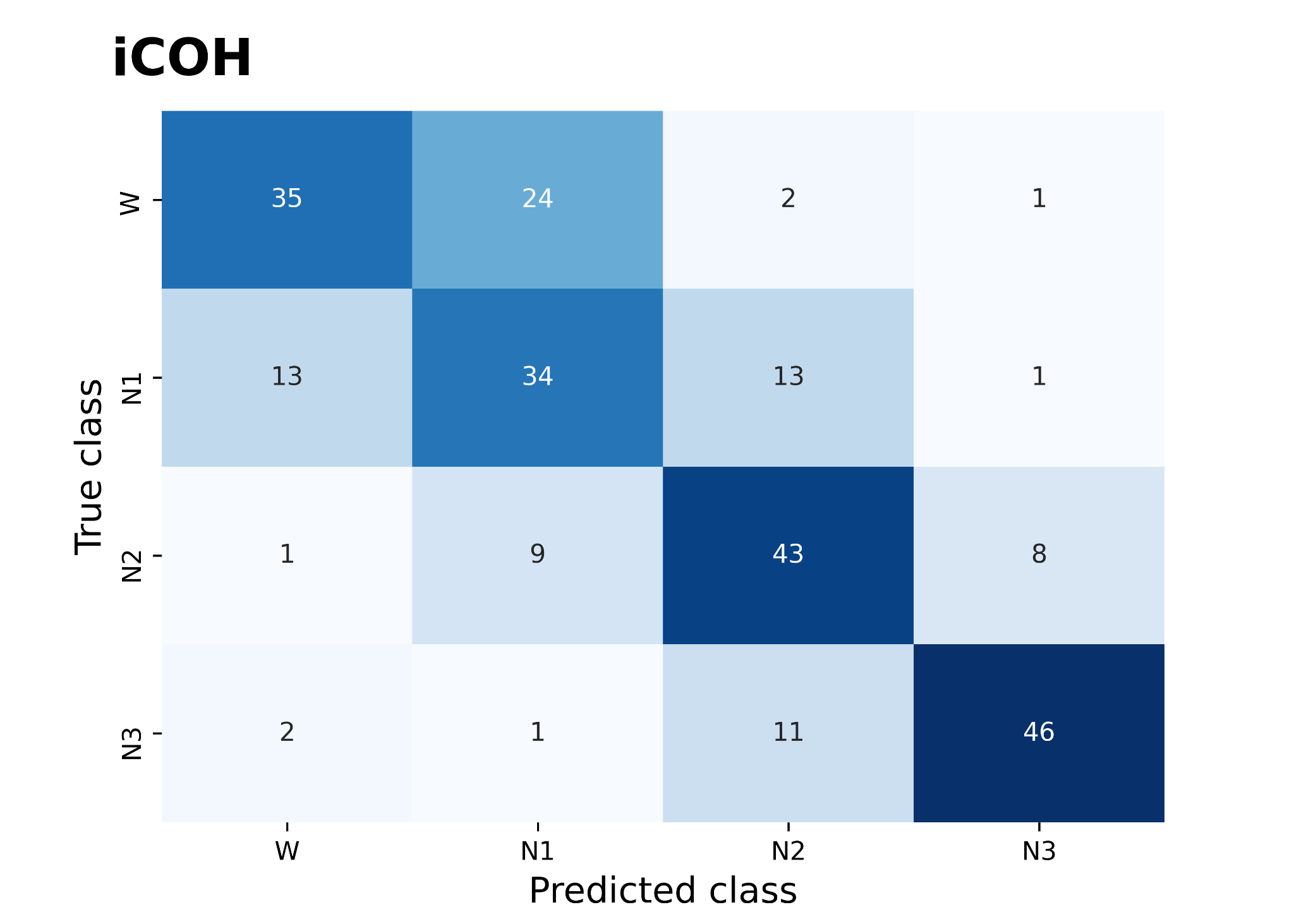 |
| --- |
|  |
| 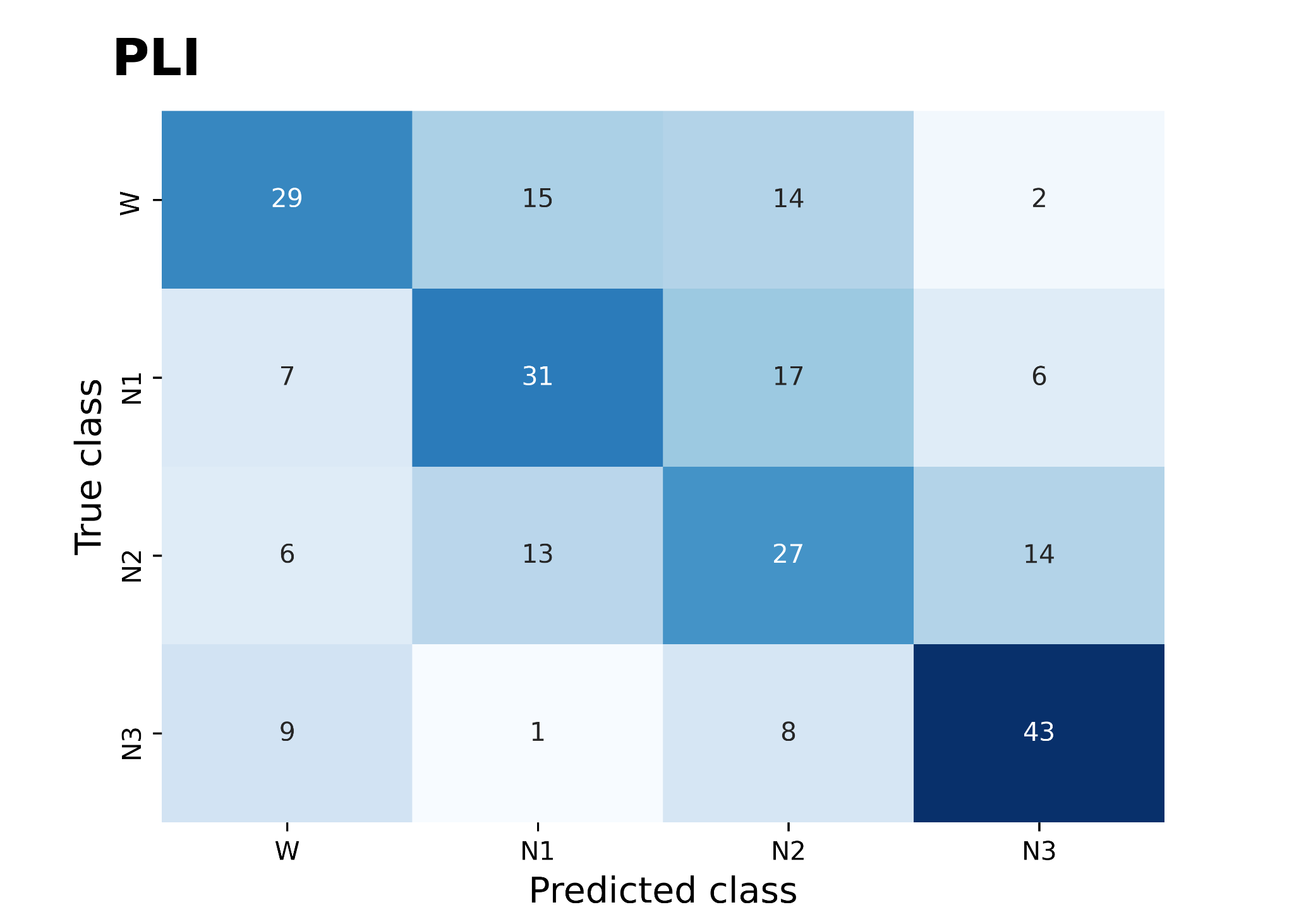 |
| *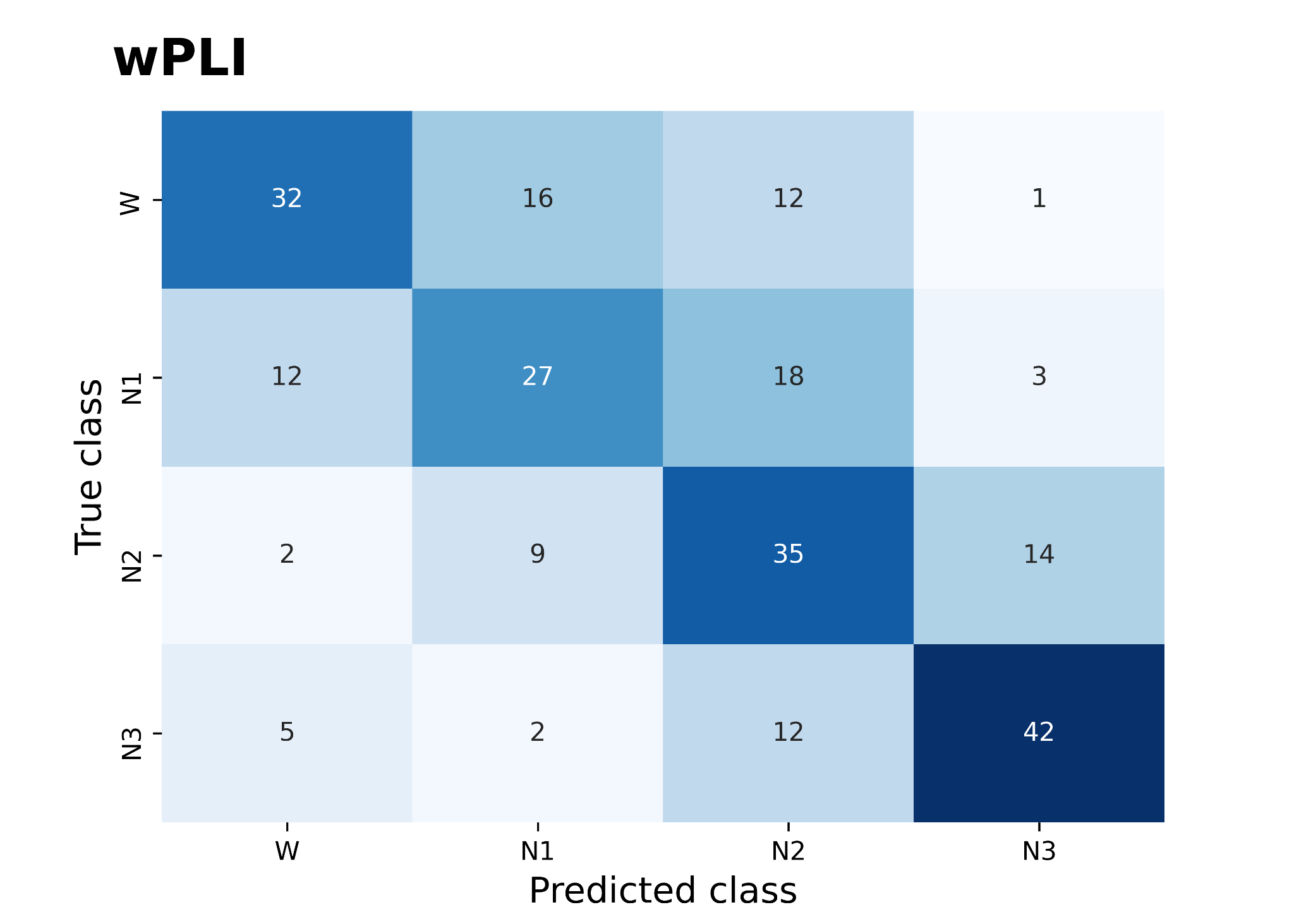* |

***Figure S2.*** *Confusion matrices for classification results based on the phase-coupling metrics iCOH, ciPLV, PLV and wPLI averaged over 30 train-test data splits. The matrices display the number of correctly classified sleep stage epochs on the diagonal. Off-diagonal elements represent incorrect classifications. AASM-based sleep scoring is set as ground truth.*

| 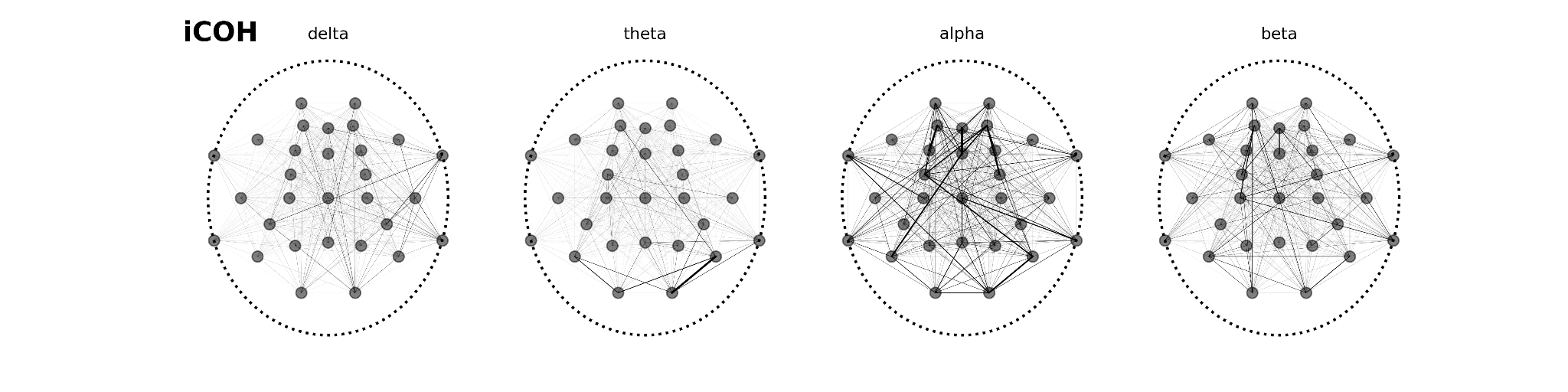 |
| --- |
| 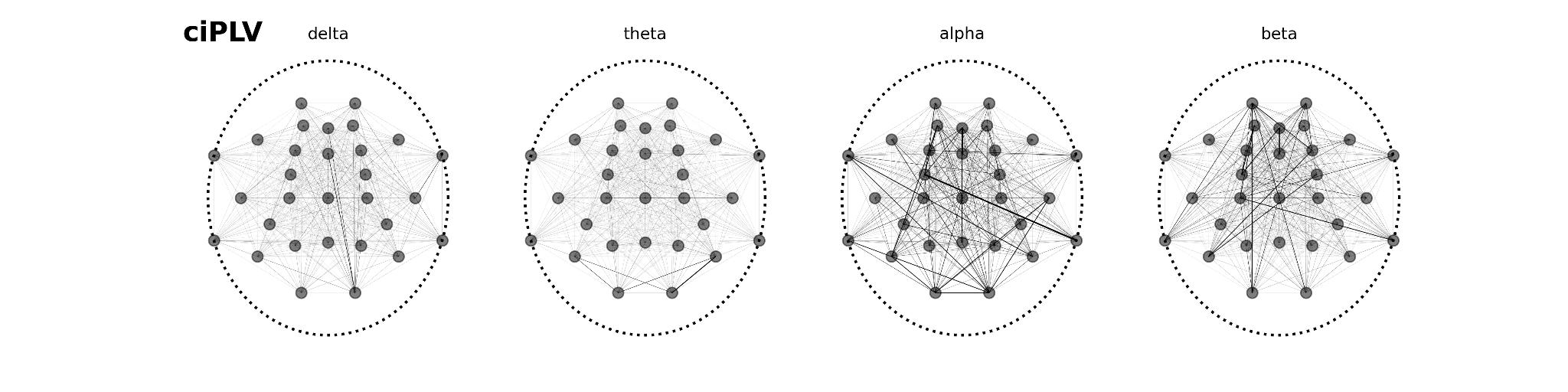 |
| 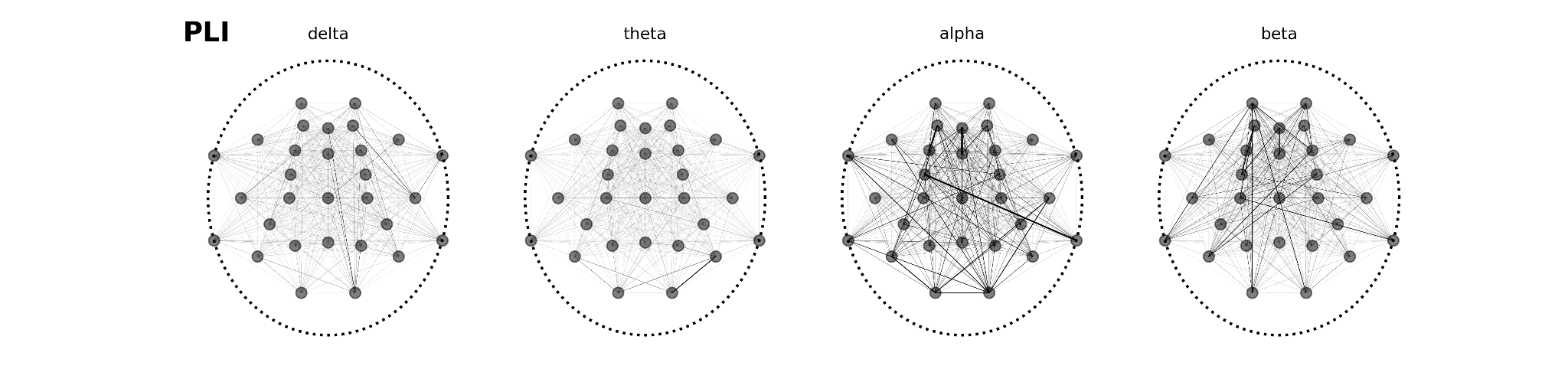 |
| 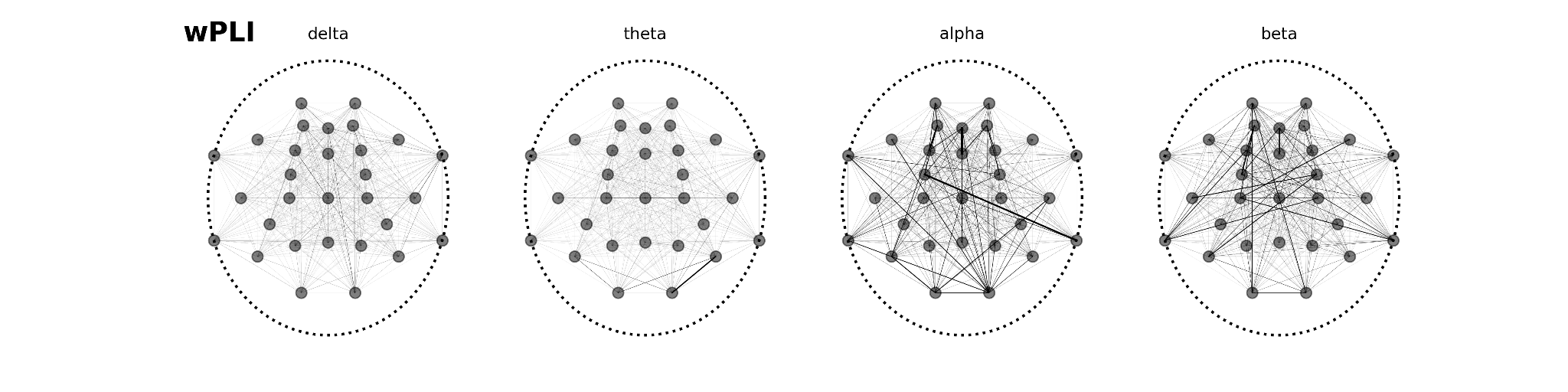 |

***Figure S3. Mean feature importances for iCOH-, ciPLV-, PLI- and wPLI-based Gradient Boosting Classifiers* (GBC)*.*** *Line width reflects the feature importance of each EEG sensor pair. Note that feature importance scores are normalized (their sum equals 1.0) in A and B, respectively, to allow visual comparison of line widths between frequency bands.*

**NREM sleep stage classification through spectral power features**

To further differentiate the influence of oscillatory power (amplitude) and phase properties on classification accuracy, we also trained Gradient Boosting Classifiers on spectral power features. We analysed the same 10 second segments from which the six phase-coupling metrics were derived. Phase-coupling was computed between 30*29/2=435 non-identical EEG channel pairs (30 EEG channels) and resulted in 4*435=1740 features for classification (4 frequency bands). Spectral power calculations resulted in 4*30=120 features (4 frequency bands x 30 EEG channels). For each 10 second segment, the power spectral density at each electrode was calculated with Welch’s method, using a Hann window of size 512 samples (2048 ms). EEG data sets were scaled to unit variance to eliminate effects introduced by inter-individual differences in absolute EEG amplitude and to focus on the relative contribution of each frequency band. Finally, the mean spectral power within each frequency band (frequency bands defined in the main text) was computed to obtain a scalar feature per electrode and per frequency band.

Classifier training followed the same principles as detailed in the main text. Briefly, we used 30 different 80/20 train/test splits on the subject level. For n=14 subjects, this was implemented using data from 11 randomly selected subjects for training, and data from the remaining 3 subjects for testing. The GBC hyperparameters ‘learning rate’, ‘n_estimators’ and ‘max_depth’ were optimised on the training data set by searching the grid defined by all hyperparameter combinations, based on the resulting classification accuracy across a five-fold cross-validation procedure. The 30 optimised GBC models were evaluated on their respective test data sets and their classification accuracies, confusion matrices, and feature importances were averaged for the final evaluation.

Results: The mean classification accuracy from spectral power features was 0.80, with a 95% confidence interval of (0.71, 0.88), the median accuracy was 0.81. The averaged confusion matrix is shown in Figure S3 below.


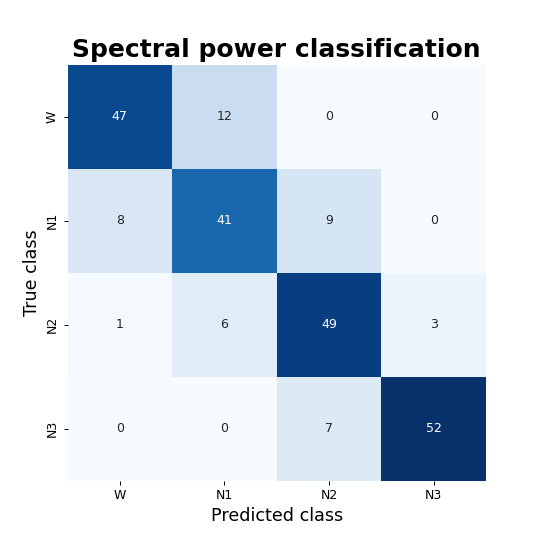


***Figure S4.*** *Confusion matrix for classification results based on spectral power, averaged over 30 train-test splits on the subject level. The matrix displays the number of correctly classified sleep stage epochs on the diagonal. Off-diagonal elements represent incorrect classifications. AASM-based sleep stages defined the ground truth. Median classification accuracy based on spectral power: 0.81 (95% CI: (0.71, 0.88)).*
